# Cardiovascular exercise mitigates reperfusion failure and persistent hypoperfusion in the thrombin model of stroke and thrombolysis

**DOI:** 10.1177/0271678X251405677

**Published:** 2026-01-16

**Authors:** William Middleham, Nadine F Binder, Julian Deseö, Robert Weber, Kirill Zolotko, Jeanne Droux, Hikari Yoshihara, Matthias T Wyss, Andreas R Luft, Bruno Weber, Daniel Razansky, Mohamad El Amki, Susanne Wegener

**Affiliations:** 1 Department of Neurology, University Hospital and University of Zürich, Zürich, Switzerland; 2 Neuroscience Center Zürich, University of Zürich, ETH Zürich, Zürich, Switzerland; 3 Department of Information Technology and Electrical Engineering, Institute for Biomedical Engineering, ETH Zürich, Zürich, Switzerland; 4 Faculty of Medicine, Institute of Pharmacology and Toxicology, University of Zürich, Zürich, Switzerland; 5 Cereneo, Center for Neurology and Rehabilitation, Vitznau, Switzerland

**Keywords:** Cardiovascular exercise, ischemic stroke, reperfusion, reperfusion failure, thrombolysis

## Abstract

Ischemic stroke remains a significant healthcare challenge, with reperfusion failure – insufficient tissue reperfusion despite successful vessel recanalization – limiting the effectiveness of recanalization treatments. However, mechanisms of reperfusion failure remain elusive, and effective treatments are lacking. This study explored whether early cardiovascular exercise could mitigate reperfusion failure. Using a rat model of ischemic stroke and thrombolysis, treadmill exercise was initiated 2 days post-stroke. Brain perfusion and functional recovery were assessed using MRI, Laser Speckle Contrast Imaging (LSCI) and functional tests. All rats treated with intravenous thrombolysis (IVT) demonstrated successful clot dissolution. However, brain perfusion only recovered to 67.6% ± 8.7% of baseline levels. Hypoperfusion persisted up to 28 days post-stroke (89.9% ± 4.8% of baseline levels). Treadmill exercise significantly mitigated reperfusion failure and chronic hypoperfusion, exhibiting substantial improvements in brain perfusion at 28 days (102.3% ± 1% of baseline levels). This resulted in smaller T2 hyperintense lesion volumes (10.4 ± 8.5 mm^3^ vs 26.7 ± 21.1 mm^3^) and enhanced functional recovery. These findings suggest that reperfusion failure transitions into chronic hypoperfusion after stroke, impeding recovery, but may be alleviated by early cardiovascular exercise, which improves vascular resilience and reperfusion recovery following ischemic stroke. Further clinical studies are needed to determine whether similar benefits can be achieved in stroke patients.

## Introduction

Current gold standard treatments for ischemic stroke focus on restoring blood flow to the brain via intravenous thrombolysis (IVT), mechanical thrombectomy, or a combination of both.^1–4^ Recanalization of the occluded vessel is crucial as it restores blood flow to the brain. However, even after successful recanalization, brain tissue may not fully reperfuse.^5^ This reperfusion failure is thought to involve several mechanisms such as clot fragmentation and distal embolization,^6–8^ neutrophil obstruction of capillaries,^9,10^ platelet activation^11,12^ and pericyte contraction.^13^ Currently, strategies to counteract reperfusion failure are lacking, but needed to improve stroke treatment outcomes. Through protective mechanisms on neuronal health and cerebral blood flow, cardiovascular exercise (CVE) could be a feasible strategy to enhance reperfusion after stroke.^14–17^

CVE is often integrated into neurorehabilitation programs to counteract deconditioning and enhance motor learning.^18–20^ CVE usually consists of treadmill exercise or bicycle ergometers and is characterized as an activity that can be maintained continuously and activates large muscle groups that use aerobic metabolism to provide energy.^21,22^ Exercise changes body homeostasis of various metabolites, such as glucose and lactate, and can increase cerebral blood flow (CBF) through changes in pCO_2_ and mean arterial blood pressure.^23,24^ However, it remains unknown if (i) CVE could counteract reperfusion failure and chronic hypoperfusion and (ii) induce clinically relevant, lasting effects on functional recovery. Our goal was to assess the extent, duration, and consequences of reperfusion failure and hypoperfusion to the subacute and chronic phases after stroke and recanalization, and to test if CVE could further enhance brain perfusion and recovery.

## Materials and methods

### Animals

Experiments were performed on Sprague Dawley rats (RjHan:SD, Janvier) between the ages of 10 and 12 weeks. Both male and female rats were included in all experimental groups, with sex distribution reported in the figure legends. Exploratory analyses comparing outcomes between males and females revealed no significant sex-specific differences across any outcome measures. A total of 28 female rats and 28 male rats were used, with sample sizes informed by prior pilot studies, aimed to detect differences in brain perfusion recovery. Sample size calculations considered estimated effect sizes and standard deviations from pilot data to achieve 80% power at a 0.05 significance level. Animals were housed in pairs under a 12-h light/dark cycle with unrestricted access to food and water. Cages were randomly assigned to control or treatment groups using a lottery method. All experiments were approved by the local veterinary authorities in Zürich (animal welfare assurance numbers ZH010/19 and ZH197/2022) and conformed to the guidelines of the Swiss Animal Protection Law, Veterinary Office, Canton of Zürich (Act of Animal Protection 16 December 2005 and Animal Protection Ordinance 23 April 2008). This study was conducted in accordance with the ARRIVE guidelines.

### Anaesthesia

Anaesthesia was induced with isoflurane at 5% in 100% oxygen (800–1000 mL/min) and maintained between 1.5% and 2.5% in a mixture of 100% oxygen and room air (100/300 mL/min, respectively). During stroke surgery and laser speckle contrast imaging (LSCI) imaging, the body temperature of the rat was monitored and maintained at 37°C using a heating pad (Homoeothermic monitor, Harvard Apparatus, USA). During MRI imaging, body temperature was maintained at 37°C using a heated water system (LAUDA Eco Silver, Germany).

### Thrombin stroke model

Focal cerebral stroke was induced using the thrombin model, adapted from previous studies in mice.^9,25,26^ The rat’s head was fixed in a stereotactic frame, and the eyes were lubricated with vitamin A ointment (Bausch + Lomb). An incision was made in the scalp along the midline axis from the left eye to the ear. A part of the left temporal muscle was cut and retracted from the skull to expose the MCA region. The skull was thinned using a dental drill (Bien Air, Osseodoc) over the left hemisphere until a thin layer of the skull remained through which the cerebral surface blood vessels were visible. A craniotomy was performed over the distal M2 segment of the MCA, and the dura was removed over the arterial branch. Stroke was induced by introducing a glass pipette (calibrated at 15 mm/µL, Assistant ref. 555/5, Hoechst, Sondheim-Rhoen, Germany) into the lumen of the MCA, at the M2 branch, followed by the injection of 3 µL of purified human alpha-thrombin (5 UI/µL, HCT-0020, Haematologic Technologies Inc., USA). After injection, the pipette remained within the MCA lumen for 10 min, after which it was removed, and the stroke LSCI recording was initiated. Based on our previous work, successful stroke induction was confirmed by an immediate drop in CBF, observed on the LSCI recording, to below 50% of baseline for at least 30 min. Any animal that did not meet this criterion was excluded from the study. The status of clot lysis or remaining occlusion of the MCA was confirmed by direct visualization of the clot through a stereomicroscope after the LSCI recording. Partial recanalization was defined as the presence of a residual clot at the M2 branch of the MCA.

Out of 64 rats, 4 were excluded due to an insufficient drop in perfusion (less than 50% of baseline for 30 min). An additional four rats died during the course of the experiments and were not included in the final analysis.

### Intravenous thrombolysis

After 30 min of stroke, thrombolysis was initiated by injecting recombinant tissue plasminogen activator (rt-PA; 10 mg/kg, Actilyse, Boehringer Ingelheim) via the tail vein (*n* = 30). As detailed by previous studies,^9,26^ 10% was initially given as a bolus injection, followed by the infusion of the remaining 90% over 45 min using a syringe pump (World Precision Instruments, USA). Rats in the control group received an equivalent volume of saline instead of rt-PA (*n* = 10).

### Laser speckle contrast imaging (LSCI)

Wide-field cortical perfusion was measured using a laser speckle contrast imaging device (moorO2FLO2, moor instruments, UK) before stroke (3 min), during the stroke (30 min) and during thrombolysis (60 min). The acquisition of perfusion images was performed at a frame rate of 1 frame/3 s. LSCI files were exported and analyzed using the moorO2Flo Image Review V1.0 software (Moor Instruments, UK), with perfusion expressed in arbitrary units based on a 256-colour palette.^27,28^ All analyses were performed by an investigator blinded to the treatment groups.

### Hypercapnic challenge

Prior to stroke and on the days post-stroke, a hypercapnic challenge was administered during the MRI protocol to assess cerebrovascular reserve in response to a vasodilatory stimulus. CBF response was measured using arterial spin labelling (ASL). An 8% CO_2_ stimulus was given for 2 min as an adjustment period, followed by initiation of the ASL sequence and an additional 5 min of 8% CO_2_. Changes in CBF in response to hypercapnia were determined by a blinded investigator through comparison of CBF maps generated from ASL images acquired before and during hypercapnia.

### Magnetic resonance imaging (MRI)

All MRI scans were performed on a 7 Tesla Bruker PharmaScan 70/16 horizontal bore small animal MR system. Respiration, heart rate and temperature were monitored using an MR-compatible animal monitoring system (Powerlab, Osensa, LabChart). MRI was performed using a ^1^H receive-only 2 × 2 rat brain surface array coil combined with a transmit volume coil (Bruker, Ettlingen, Germany). T2-weighted imaging was acquired using a TurboRARE sequence to determine lesion volume, with infarct areas defined by cortical hyperintensity. Lesion volumes were assessed at days 1, 4, 14 and 28 post-stroke by manually delineating hyperintense areas on each slice and calculating the total lesion volume by summing lesion areas and multiplying by slice thickness. To account for post-ischemic edema, a validated indirect method was used to calculate T2 corrected hyperintense lesion volumes by multiplying the raw lesion volume by the ratio of the contralateral to ipsilateral hemisphere volume:

$$\begin{array}{l} \mathrm{Corrected}\;\mathrm{lesion}\;\mathrm{volume}=\mathrm{Raw}\;\mathrm{lesion}\;\mathrm{volume}\times \\ \frac{\mathrm{Contralateral}\;\mathrm{hemisphere}\;\mathrm{volume}}{\mathrm{Ipsilateral}\;\mathrm{hemisphere}\;\mathrm{volume}} \end{array}$$

Hemisphere volumes were determined by manually outlining and summing the cross-sectional area of each hemisphere on the same slices used for T2 hyperintense lesion volume analysis. T2 hyperintense lesion volumes were manually calculated using ImageJ (NIH). ASL imaging was used to evaluate CBF, with CBF maps generated from raw ASL data using Matlab. Analysis of all MRI data was performed by an investigator who was blinded to group allocation.

### Treadmill training protocol

For CVE, all rats (both in the exercise and no-exercise groups) completed a three-day treadmill training period before stroke or sham stroke surgery to accommodate the treadmill training environment. The training involved placing the rats on the treadmill (Five lane touchscreen treadmill, Panlab, Harvard Bioscience Inc., product code: 760895) for 30 min at speeds of 14, 15 and 16 cm/s on training days 1, 2 and 3, respectively. The treadmill slope remained at 0°. Post-stroke treadmill exercise commenced on day 2 and continued for 2 weeks with 5 exercise sessions per week, lasting 30 min at 17 cm/s (stroke and treadmill *n* = 10, sham stroke and treadmill *n* = 5). Rats in the no-treadmill exercise groups were placed on the treadmill for 30 min without the treadmill being turned on (stroke and no treadmill *n* = 10, sham stroke and no treadmill *n* = 4).

### Adhesive tape removal test

The adhesive tape removal test was used to assess sensorimotor function, with testing and analysis performed by an investigator blinded to treatment groups.^29^ Two strips of tape were applied to both forepaws of the rat in a random order. The time taken for the rat to first contact the tape (sensory) and the time taken to remove the tape (motor) were recorded. Each session included three trials and results were averaged. Before stroke, all rats were trained to remove the tape within 10 s.

### Novel object recognition test

The novel object recognition test (NOR) was used to assess cognitive function after stroke.^30,31^ One day before the test, rats explored the arena freely for 30 min. For analysis, the discrimination index was calculated as described previously.^31^ Rats were placed in an arena (100 cm × 50 cm × 40 cm, Noldus) with two identical objects and allowed to explore freely and undisturbed for 5 min (familiarisation phase). After 3 h (rest phase), during which the arena was cleaned and one object replaced with a new one, rats were returned to the arena for another 5-min exploration phase. During the familiarisation and exploration phases, rats were recorded with a GigE Basler monochrome camera (Noldus). Videos were analysed using EthoVision XT (Noldus) by an investigator blinded to all treatment and control groups. Nose, body and tail points were automatically tracked, and all tracking was visually inspected and manually corrected. Exploration of an object was defined as the nose point being within 9 cm of the centre of the object or touching the object.

### Immunohistochemistry

Rats were anaesthetised with pentobarbital and transcardially perfused with PBS solution. Brains were extracted, post-fixed in 4% PFA overnight, and transferred to 30% sucrose solution. Brains were sectioned into 60 μm slices using a microtome with a vibrating blade (MICROM HM 650 V, Thermo Fischer Scientific).

For the immunofluorescent protocol, slices were incubated overnight at 4°C in a solution of 0.25% Triton X-100, with 3% normal donkey serum in 0.1 M Tris Buffered saline (TBS). Primary antibodies were applied and incubated for 3 days at 4°C, followed by thorough washing with TBS and incubation with secondary antibodies for 2 h at room temperature. DAPI (1:10000; D9542; Merck) was used to stain nuclei. Slices were then mounted on slides with a fluorescent mounting medium (Agilent Technologies, S302380).

Neurons were stained with mouse anti-NeuN (1:1000; MAB377; Merck), astrocytes were stained with rabbit anti-GFAP (1:1000; G9269; Sigma-Aldrich), endothelial cells on vessels were stained with mouse anti-RECA-1 (1:400; MCA970R; BIO-RAD), microclots and BBB damage were stained with sheep anti-fibrinogen (1:300; LS-B2573; LSBio). Alexa Fluor647 AffiniPure donkey anti-rabbit IgG (H + L; 1:400; 711-605-152; JacksonImmuno), Alexa Fluor647 AffiniPure donkey anti-sheep IgG (H + L; 1:400; 713-605-147; JacksonImmuno), Alexa Fluor488 donkey anti-mouse IgG (H + L; 1:400; 715-545-151; JacksonImmuno) were used as secondary antibodies.

Immunohistochemical analyses were conducted by an investigator who was blinded to group assignments. *Z*-stacks (40–50 μm) were acquired at 10× or 20× magnification using a Zeiss LSM 800 confocal laser scanning microscope. ROIs were selected from the peri-infarct and corresponding contralateral areas. For sham stroke animals, ROIs were taken from comparable cortical areas. Image analysis was performed using ImageJ (NIH) and the experimenter was blinded to the experimental groups. NeuN-positive cells were counted for selective neuronal death analysis, GFAP signal intensity measured reactive astrogliosis, microclots were counted manually, and extravascular fibrinogen signal intensity assessed BBB leakage. Vessel density was analyzed using the vessel analysis plugin in ImageJ, where: vascular density = vessel area/total area * 100.

### Statistical analysis

Statistical analysis was performed using GraphPad Prism (version 10.2.0; GraphPad Software La Jolla, CA, USA). Data in all groups was tested for normality using the D’Agostino-Pearson omnibus normality test. For comparisons between two groups, significance (*p* < 0.05) was determined using the two-tailed Mann–Whitney *U* test. For experiments involving repeated measurements across treatment groups and time, two-way repeated measures ANOVA was applied to evaluate main effects of treatment, time, and their interaction, followed by Sidak’s post hoc tests for pairwise comparisons at individual timepoints. See Supplemental Table 1 for full statistical results. Correlation analysis was calculated using Spearman’s rank correlation coefficient. Results are presented as mean ± SD (standard deviation) or median (interquartile range) in violin plots, with details of statistical tests and group sizes (*n*) provided in the figure legends.

## Results

### IVT in the thrombin model of stroke in rats induces full recanalization but incomplete reperfusion

**Figure 1.**
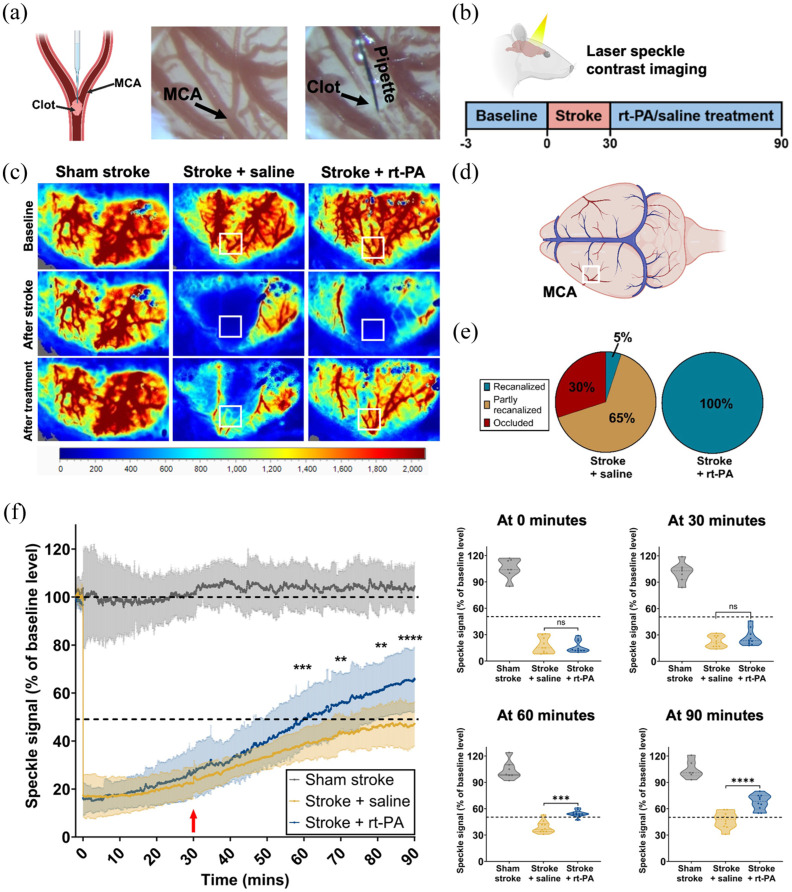
Reperfusion failure in the thrombin model of stroke in rats. (a) A graphic of the injection of thrombin and clot formation in the MCA branch (left). An image of the M2 branch of the MCA before (middle) and after clot formation (right). (b) The experimental timeline of LSCI imaging includes three recording periods: a 3-min baseline recording, a 30-min stroke recording, and a further 60-min recording capturing thrombolysis. These time points were chosen to capture the dynamic changes in perfusion following stroke induction and saline/rt-PA treatment. (c) Representative LSCI images of the three recording periods (baseline, stroke, treatment) for sham stroke, stroke + saline and stroke + rt-PA animals. The ROI for LSCI analysis is shown as a white box and was placed over the MCA, based on anatomy extracted from the baseline LSCI image. The scale bar shows CBF (a.u.). (d) A graphic representing the whole rat brain and major vessels with the location of the ROI for laser speckle analysis. (e) Quantification of the rate of MCA recanalization at the M2 branch 90 min after stroke induction for stroke + saline (left, *n* = 10, female = 5, male = 5) and stroke + rt-PA (right, *n* = 10, female = 5, male = 5) groups. (f) Graph left: Analysis of LSCI recordings of the region of interest in sham stroke, stroke + saline and stroke + rt-PA rats. The red arrow indicates the starting time of the administration of saline or rt-PA. Sham stroke *n* = 7 (female = 3, male = 4), stroke + saline *n* = 10 (female = 5, male = 5), stroke + rt-PA *n* = 10 (female = 5, male = 5). Graphs right: Distribution of speckle signal changes within MCA ROI at individual time points of 0, 30, 60 and 90 min during the LSCI recording for animals in sham stroke (*n* = 7, female = 3, male = 4), stroke + saline (*n* = 10, female = 5, male = 5) and stroke + rt-PA (*n* = 10, female = 5, male = 5) groups. Treatment group differences (stroke + saline vs stroke + rt-PA) over time were analyzed by two-way repeated measures ANOVA, followed by Sidak’s post hoc test. **p < 0.01. ***p < 0.001. ****p < 0.0001.

To investigate whether reperfusion failure occurs in the rat thrombin model of stroke, we utilized laser speckle contrast imaging (LSCI), monitoring cortical perfusion in the ipsilateral hemisphere over a period of 90 min (Figure 1(c)).

Thrombin injection into the left middle cerebral artery (MCA), M2 segment, successfully induced clot formation leading to the occlusion of the targeted vessel (Figure 1(a)). This resulted in a significant drop in perfusion within the cortical MCA area reducing to 17.4% ± 8.9% for the saline-treated group and 16.2% ± 6.8% for the rt-PA-treated animals (Figure 1(f)). Thrombolysis was initiated by administering rt-PA 30 min after the onset of stroke to dissolve the clot in situ.^9,25,26^

Clot lysis at 90 min post-stroke induction was achieved in 100% of rt-PA-treated rats versus 5% of saline-treated rats (Figure 1(e)). Spontaneous reperfusion (CBF higher than 50% of baseline) occurred in a few rats (*n* = 4, 40%) within the saline-treated group, but average levels remained below 50% of baseline perfusion values (stroke + saline: 47.2% ± 9.1%) at the end of the LSCI recording (Figure 1(c) and (f)). Treatment with rt-PA caused significant reperfusion compared to saline treatment (rt-PA: 67.6% ± 8.7% vs saline: 47.2% ± 9.1%, *p* < 0.0001) at 90 min after stroke (Figure 1(c) and (f)). Despite achieving a 100% recanalization rate, the rt-PA-treated group only recovered to 67.6% ± 8.7% of baseline levels in the MCA territory. These findings highlight the occurrence of reperfusion failure in rats subjected to the thrombin model of stroke. Notably, this observation aligns with our previous findings in the same model using mice.^9^

### Reperfusion failure persists through 7 days post-stroke

**Figure 2.**
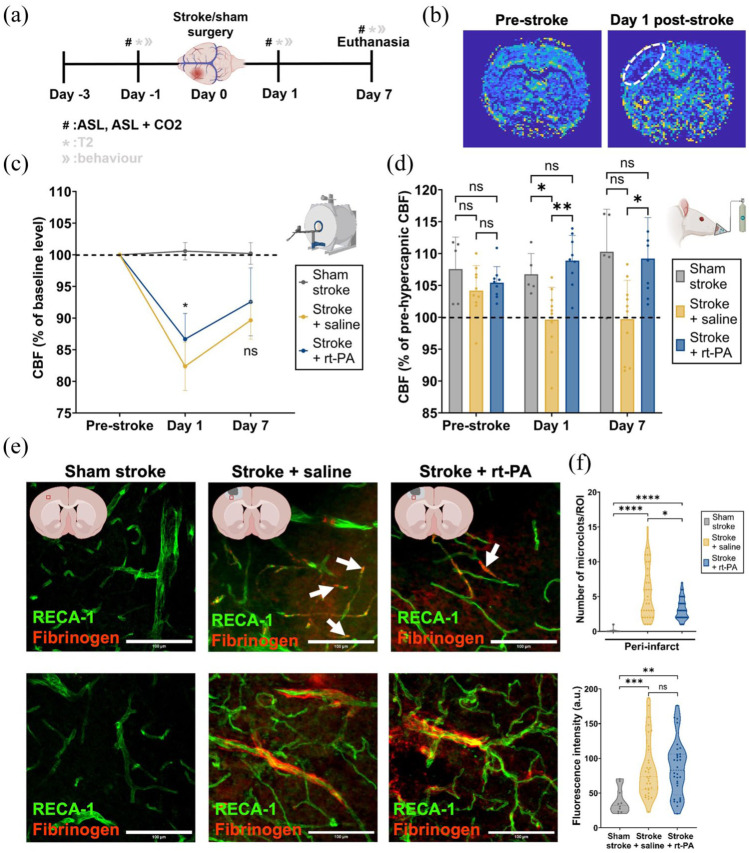
Assessment of cerebral perfusion, vascular reactivity, BBB integrity, and microclot formation following ischemic stroke and treatment with rt-PA. (a) Experimental timeline showing the days when the stroke surgery, perfusion imaging, hypercapnic challenge and euthanasia were performed. (b) Representative calculated CBF maps from ASL imaging showing perfusion in the whole brain pre-stroke (left) and on day 1 post-stroke (right). The lesion area is highlighted by the white dotted line. (c) CBF change, calculated from pre-stroke levels, on days 1 and 7 post-stroke. Sham stroke *n* = 7 (female = 3, male = 4), stroke + saline *n* = 10 (female = 5, male = 5), stroke + rt-PA *n* = 10 (female = 5, male = 5). Group differences (stroke + saline vs stroke + rt-PA) over time were analyzed by two-way repeated measures ANOVA, followed by Sidak’s multiple comparisons test. (d) Relative CBF change upon a hypercapnic challenge with 8% CO_2_ pre-stroke, and on days 1 and 7 post-stroke. Sham stroke *n* = 7 (female = 3, male = 4), stroke + saline *n* = 10 (female = 5, male = 5), stroke + rt-PA *n* = 10 (female = 5, male = 5). Data were assessed using two-way repeated measures ANOVA comparing all three groups with Sidak’s post hoc test. (e) Representative immunofluorescent images showing examples of microclots (first row), and BBB leakage (second row), for the sham stroke group (first column), stroke + saline group (second column), and stroke + rt-PA group (third column). ROIs are taken from the peri-infarct area, and an example location of the ROI can be seen in the graphics at the top of each column. (f) Top: Quantification of the number of microclots/ROI observed for each group in the peri-infarct area on day 7 post-stroke. Sham stroke n = 5 (female = 2, male = 3), slices = 10, ROIs = 20; stroke + saline n = 8 (female = 4, male = 4), slices = 16, ROIs = 32; stroke + rt-PA n = 8 (female = 4, male = 4), slices = 16, ROIs = 32. Bottom: BBB leakage represented by fluorescence intensity of fibrinogen in the peri-infarct area for each group on day 7 post-stroke. Sham stroke *n* = 5 animals (female = 2, male = 3); stroke + saline *n* = 8 animals (female = 4, male = 4); stroke + rt-PA *n* = 8 animals (female = 4, male = 4). Group comparisons were made using the two-tailed Mann–Whitney *U* test. *p < 0.05. **p < 0.01. ***p < 0.001. ****p < 0.0001.

To investigate whether reperfusion failure extends beyond the acute phase of stroke, we examined whole-brain perfusion using arterial spin labelling (ASL) MRI at days one and seven post-stroke (Figure 2(a) and (b)). A T2 sequence was acquired in the same orientation as the ASL images to ensure precise selection of the ROI within the T2 hyperintense lesion area. Both stroke groups exhibited significant reduction in perfusion within the T2 hyperintense area on days 1 and 7 (Figure 2(c)). Animals treated with rt-PA had better-maintained perfusion on day 1 (stroke + rt-PA: 88.5% ± 4.1%, stroke + saline: 83.4% ± 3.8%, *p* = 0.0257; Figure 2(c)). However, by day seven, no significant difference in perfusion was observed between the rt-PA and saline-treated groups, with both remaining below baseline levels (stroke + saline: 89.7% ± 3%, stroke + rt-PA: 93.5% ± 5.4%; Figure 2(c)). These findings suggest that reperfusion failure persists for at least 7 days post-stroke, even with rt-PA treatment and successful recanalization. Due to the larger ROI, involving deeper areas, and methodological differences, changes in ASL-derived CBF are different from changes in LSCI signal (Figure 2(c)).

To determine whether reperfusion failure was due to impaired large vessel function, cerebrovascular reactivity was assessed using MRI. ASL imaging was performed in combination with a 5-min 8% CO_2_ challenge on days 1 and 7 post-stroke. An expected increase in perfusion upon the hypercapnic stimulus was observed in the sham stroke group on all days. Saline-treated rats showed a significantly diminished response to the hypercapnic stimulus on both days (day 1: *p* = 0.0004, day 7: *p* = 0.0044) compared to rt-PA treated animals, which exhibited a similar response to sham stroke animals on both days post-stroke (Figure 2(d)). While in saline-treated rats, cerebrovascular reactivity was clearly diminished, rt-PA treated rats did not show a dysfunction in the large pial vascular network, suggesting that the lack of reperfusion in the recanalized ischemic area is not due to a loss of cerebrovascular reactivity but rather attributable to reperfusion failure of the microvascular bed.

Next, we wanted to explore the effect of stroke on the brain microvasculature. Using immunofluorescence, we examined the presence of microclots, and the BBB integrity in the peri-infarct area on day 7 post-stroke (Figure 2(e)). Quantification of intravascular microclots showed an increased number of microclots in small vessels in the peri-infarct area (Figure 2(f)). Saline-treated animals had significantly more microclots within the peri-infarct area, 5.6 ± 3.8/ROI, than both sham stroke and rt-PA-treated animals (0.1 ± 0.3/ROI, *p* < 0.0001 and 3.2 ± 1.6ROI, *p* = 0.0171, respectively; Figure 2(f)). Extravascular fibrinogen fluorescence intensity indicated significant BBB damage in the peri-infarct area for both stroke + saline and stroke + rt-PA groups (Figure 2(e) and (f)). Altogether, these findings indicate a significant microvascular failure characterized by an increased presence of microclots and BBB damage following stroke. Our experiments demonstrated that despite successful recanalization of the MCA with rt-PA, microvascular reperfusion failure persisted for at least 7 days post-stroke.

### Reperfusion failure is associated with an incomplete recovery of function

**Figure 3.**
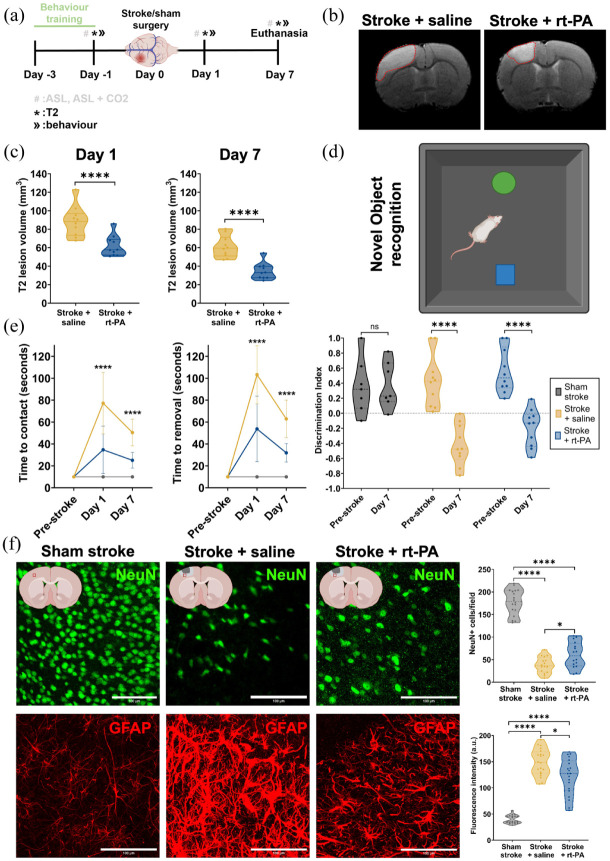
Evaluation of functional outcomes and tissue damage following ischemic stroke and rt-PA treatment. (a) Experimental timeline showing the timings of the behavioural training, pre-behaviour and MRI, the stroke surgery, post-stroke MRI and behaviour and euthanasia. (b) Representative T2 hyperintense signal on day 1 for the stroke + saline (left) and the stroke + rt-PA (right) groups. The hyperintense signals are outlined with red dotted lines. (c) The differences in the calculated T2 hyperintense signal volumes between the stroke + saline group and stroke + rt-PA group on days 1 (left) and 7 (right). Stroke + saline *n* = 10, stroke + rt-PA *n* = 10. Changes in T2 hyperintense lesion volumes were assessed using two-way repeated measures ANOVA with Sidak’s post hoc test. (d) A graphic showing the exploration phase of the novel object recognition test. (e) Average time to contact (left) and time to removal (middle) for each group, pre-stroke and on days 1 and 7. Sham stroke *n* = 7 (female = 3, male = 4), stroke + saline *n* = 10 (female = 5, male = 5), stroke + rt-PA *n* = 10 (female = 5, male = 5). (Right) The calculated discrimination index from the NOR test for each group pre-stroke and on day 7 post-stroke. Sham stroke *n* = 7 (female = 3, male = 4), stroke + saline *n* = 10 (female = 5, male = 5), stroke + rt-PA *n* = 10 (female = 5, male = 5). Behavioural outcomes were analyzed by two-way repeated measures ANOVA (comparing stroke + saline vs stroke + rt-PA) with Sidak’s post hoc comparisons. (f) Representative immunofluorescent images showing selective neuronal death and reactive astrogliosis in the periinfarct area of the stroke + saline and stroke + rt-PA groups. NeuN-positive cells are shown in the first row and GFAP-positive cells are shown in the second row. Sham stroke is represented in the first column, stroke + saline is represented in the second column, and stroke + rt-PA is represented in the third column. ROIs are taken from the peri-infarct area and an example location of the ROI can be seen in the graphics at the top of each column. (Top right) Calculation and comparison of NeuN positive cells in the peri-infarct area for all groups. Sham stroke *n* = 5 animals (female = 2, male = 3); stroke + saline *n* = 8 animals (female = 4, male = 4); stroke + rt-PA *n* = 8 animals (female = 4, male = 4). (Bottom right) Reactive astrogliosis shown as fluorescence intensity of GFAP signal in the peri-infarct area on day 7 post-stroke. Stroke + saline *n* = 8 animals (female = 4, male = 4); stroke + rt-PA *n* = 8 animals (female = 4, male = 4). Data were compared using two-tailed Mann–Whitney *U* test. *p < 0.05. ****p < 0.0001.

Next, we examined infarct size as well as sensorimotor, and cognitive function in the days following stroke. Animals were monitored on days 1 and 7 post-stroke using MRI and behavioural tests, including the adhesive removal test and the novel object recognition test, following sham stroke, stroke + saline or stroke + rt-PA (Figure 3(a)). T2-weighted imaging was employed to assess vasogenic edema after stroke. On day 1, stroke resulted in average T2 signal of 86.5 ± 16.4 mm^3^, which decreased to 61.157.4 ± 11.7 mm^3^ by day 7. Thrombolysis with rt-PA significantly reduced the T2 hyperintense lesion compared to saline-treated animals on both day 1 (61.8 ± 11.3 mm^3^, *p* < 0.0001), and day 7 (34.5 ± 8.9 mm^3^, *p* < 0.0001) post-stroke (Figure 3(b) and (c)). T2 hyperintensity volumes for both groups decreased from day 1 to 7 (Figure 3(c)).

Ischemia resulted in significant sensorimotor deficits, as demonstrated by prolonged times to contact and remove the tape on the affected side during the adhesive removal test conducted on day 1 (Figure 3(e)). On day 1, rats that received rt-PA treatment had better sensorimotor scores compared to saline-treated rats (time to contact: 34.7 ± 21.7 s vs 77.2 ± 28.1 s, *p* < 0.0001; time to removal: 53.7 ± 29.9 s vs 103.3 ± 26.5 s, *p* < 0.0001; Figure 3(e)).

This effect was again observed on day 7 where rats in the rt-PA treated group showed improved sensorimotor scores compared to saline treated rats (time to contact: 25.1 ± 7.2 vs 50.4 ± 12.2 s, *p* < 0.0001; time to removal: 31.9 ± 8.4 s vs 62.9 ± 17.1 s, *p* < 0.0001; Figure 3(e)). These results indicate that sensorimotor function improved over time across all animals, but that rt-PA treated rats consistently outperformed saline treated animals. Furthermore, stroke caused significant cognitive deficits (Figure 3(e)). Both saline and rt-PA-treated rats had lower discrimination index scores on day 7 compared to their pre-stroke scores, which indicated that the stroke affected their ability to distinguish objects in the NOR test (Figure 3(d) and (e)).

To further investigate IVT effects on neuronal damage and reactive astrogliosis, immunofluorescence markers were used to stain mature neurons (neuronal nuclear protein: NeuN) and reactive astrocytes (glial fibrillary acidic protein: GFAP) in the peri-infarct area on day 7 post-stroke (Figure 3(f)). IVT rescued a significant number of neurons (60.3 ± 28.2 vs 38.44 ± 17.9, *p* = 0.0252) and reduced reactive astrogliosis (121.91 ± 34.4 vs 148.73 ± 25.3, *p* = 0.0227) in the peri-infarct area compared to saline-treated rats (Figure 3(f)).

### Cardiovascular exercise mitigates reperfusion failure and persistent hypoperfusion post-stroke

**Figure 4.**
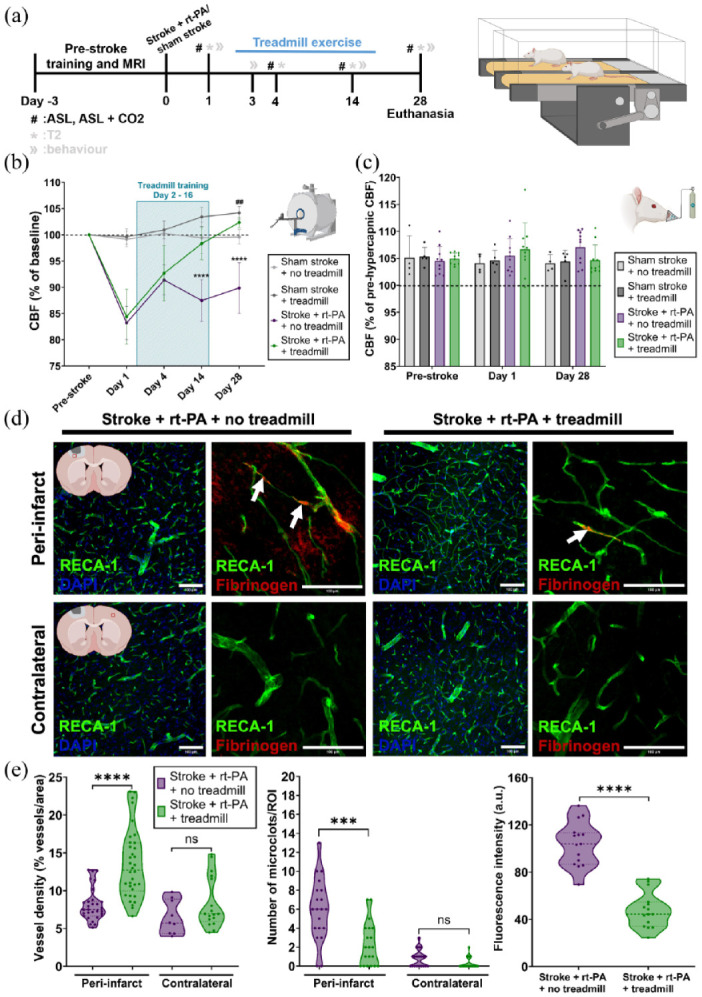
Impact of post-stroke CVE on cerebral perfusion, vascular health and microclot formation. (a) Left: Experimental timeline showing the pre-stroke treadmill training, stroke/sham stroke surgery, treadmill exercise period, all ASL perfusion measurements and day of euthanasia. Right: Graphic representation of the treadmill exercise setup. (b) Relative CBF change, calculated from pre-stroke levels, on days 1, 4, 14 and 28 post-stroke. Sham stroke + treadmill *n* = 5 (female = 3, male = 2), sham stroke + no treadmill *n* = 4 (female = 2, male = 2), stroke + rt-PA + treadmill *n* = 10 (female = 5, male = 5), stroke + rt-PA + no treadmill *n* = 10 (female = 5, male = 5). Perfusion changes were analyzed using two-way repeated measures ANOVA comparing (*) stroke + rt-PA + no treadmill versus stroke + rt-PA + treadmill and (#) sham stroke + no treadmill versus sham stroke + treadmill with Sidak’s post hoc test. (c) Relative CBF change upon a hypercapnic challenge with 8% CO_2_ pre-stroke and on days 1 and 28 post-stroke. Sham stroke + treadmill *n* = 5 (female = 3, male = 2), sham stroke + no treadmill *n* = 4 (female = 2, male = 2), stroke + rt-PA + treadmill *n* = 10 (female = 5, male = 5), stroke + rt-PA + no treadmill *n* = 10 (female = 5, male = 5). Cerebrovascular reactivity was assessed by two-way repeated measures ANOVA comparing either stroke + rt-PA + no treadmill vs. stroke + rt-PA, + treadmill or sham stroke + no treadmill versus sham stroke + treadmill, followed by Sidak’s post hoc test. (d) Representative immunofluorescent images of vessel density, microclots and BBB damage in the peri-infarct and contralateral areas for the stroke + rt-PA + no treadmill and stroke + rt-PA + treadmill groups. ROIs in the peri-infarct areas are shown in the first row and ROIs in the contralateral areas are shown in the second row. Graphics showing an example of the ROI (red box) location is shown in the graphics at the beginning of each row. Vessel density is shown in the first and third columns. Microclots and BBB damage are shown in the second and fourth columns. White arrows show microclots. (e) Left: Calculated vessel density for the peri-infarct and contralateral ROIs for the stroke + rt-PA + treadmill and stroke + rt-PA + no treadmill groups on day 28 post-stroke. Stroke + rt-PA + treadmill peri-infarct: *n* = 5 (female = 3, male = 2), slices *n* = 15, ROIs *n* = 30, contralateral: *n* = 5 (female = 3, male = 2), slices = 15, ROIs *n* = 15; stroke + rt-PA + no treadmill peri-infarct: *n* = 5 (female = 3, male = 2), slices *n* = 15, ROIs *n* = 30, contralateral: *n* = 5 (female = 3, male = 2), slices = 15, ROIs *n* = 15. Middle: Number of microclots/ROI in the peri-infarct and contralateral areas for stroke + rt-PA + treadmill and stroke + rt-PA + no-treadmill groups on day 28 post-stroke. Stroke + rt-PA + treadmill peri-infarct: *n* = 5 (female = 3, male = 2), slices *n* = 20, ROIs *n* = 20, contralateral: *n* = 5 (female = 3, male = 2), slices *n* = 20, ROIs *n* = 20; stroke + rt-PA + no treadmill peri-infarct: *n* = 5 (female = 3, male = 2), slices *n* = 20, ROIs *n* = 20, contralateral: *n* = 5 (female = 3, male = 2), slices *n* = 20, ROIs *n* = 20. Right: BBB leakage represented by fluorescence intensity of fibrinogen in the peri-infarct area for stroke + rt-PA + treadmill and stroke + rt-PA + no treadmill groups on day 28 post-stroke. Stroke + rt-PA + treadmill: *n* = 5 (female = 3, male = 2), slices *n* = 15, ROIs *n* = 15; stroke + rt-PA + no treadmill: *n* = 5 (female = 3, male = 2), slices *n* = 15, ROIs *n* = 15. Group comparisons were made using the two-tailed Mann–Whitney *U* test. ^##^*p* < 0.01. ***p < 0.001. ****p < 0.0001.

After confirming that reperfusion failure persists for at least 7 days post-stroke, we sought to explore potential treatment strategies to mitigate this effect. First, we investigated the effects of treadmill exercise on perfusion and vascular integrity (Figure 4(a)). Ten rats underwent treadmill exercise, after stroke and thrombolysis, for 2 weeks (days 2–16), with 30-min sessions 5 times a week.^32^ Perfusion was measured with ASL MRI on days 1, 4, 14 and 28 (Figure 4(a)). Consistent with our previous findings (Figure 2(c)), tissue perfusion in the ischemic area decreased to 84.4 ± 5.2% (stroke + rt-PA + treadmill exercise group) and 83.2 ± 3.2% (stroke + rt-PA + no treadmill exercise group) on day 1 (Figure 4(b)). Perfusion remained below pre-stroke levels on day 4 (stroke + rt-PA + treadmill exercise group: 92.7 ± 5.3%; stroke + rt-PA + no treadmill exercise group: 91.4 ± 2.8%; Figures 2(c) and 4(b)). Perfusion within the ischemic area of non-exercising rats remained below pre-stroke levels at 87.5 ± 4% and 89.9 ± 4.8% on days 14 and 28, respectively (Figure 4(b)). However, rats treated with cardiovascular exercise, exhibited a steady increase in perfusion from day 4 (92.7 ± 5.3%) to day 14 (98.3 ± 3.1%) and day 28 (102.3 ± 1%; Figure 4(b)). Post hoc analysis confirmed that animals in the treadmill exercise group had significantly higher perfusion than animals in the no treadmill exercise group on both day 14 (*p* < 0.0001) and 28 (*p* < 0.0001). Notably, even after treadmill exercise ended on day 16, perfusion continued to improve up to day 28, suggesting a sustained positive impact of cardiovascular exercise on perfusion (Figure 4(b)). Additionally, an overall perfusion enhancement of treadmill exercise was also evident in the sham stroke + treadmill exercise group, with cortical perfusion levels (an area resembling the usual lesion area in the stroke model) reaching 104.2 ± 1.2% on day 28, significantly higher than in sham stroke + no treadmill exercise rats (99.6 ± 1.4%, *p* = 0.0019; Figure 4(b)).

To elucidate the mechanisms underlying the effect of CVE on reperfusion failure, we measured large vessel reactivity (up to 28 days post-stroke), to a CO_2_ challenge using MRI-ASL imaging of the infarct area. Both treadmill and no treadmill groups showed normal responses to the hypercapnic challenge on day 1 after stroke (Figures 2 and 4(c)). The response to hypercapnia remained consistent into the sub-acute stage with no significant differences between treatment groups on day 28 (Figure 4(c)).

Next, we studied vessel density, microclot number and BBB damage in the peri-infarct and contralateral areas after treadmill exercise (Figure 4(d) and (e)). Strikingly, in animals that had undergone treadmill exercise, vessel density was significantly higher up to day 28 (13.5 ± 4.5%/ROI, *p* < 0.0001) in the peri-infarct area (Figure 4(e)) compared to no treadmill group, suggesting that treadmill exercise promotes angiogenesis in the sub-acute stage of stroke. Additionally, treadmill exercise resulted in fewer microclots per ROI compared to the no treadmill exercise group (2.3 ± 2.3 vs 5.95 ± 3.3/ROI, *p* = 0.0004) and reduced BBB damage (47.8 ± 15 vs 103.8 ± 18.6 fluorescence intensity/ROI, *p* < 0.0001; Figure 4(d) and (e)).

### Thrombolysis combined with post-stroke cardiovascular exercise results in significantly improved functional outcomes

**Figure 5.**
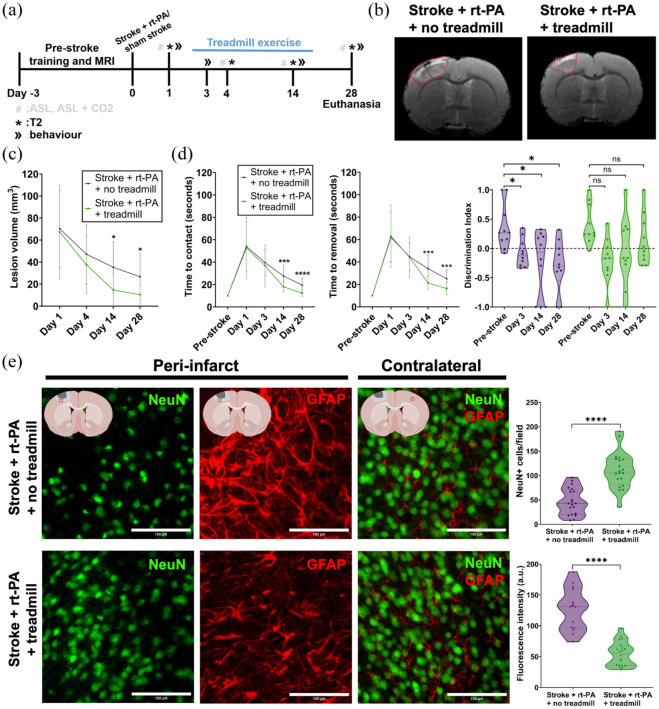
Monitoring of post-stroke CVE effects on functional outcomes and neuronal survivability. (a) Experimental timeline detailing pre-stroke training and MRI, stroke surgery, MRI and behaviour measurements, and day of euthanasia. (b) Representative images of the T2 hyperintense signal for both stroke + rt-PA + no treadmill (left) and stroke + rt-PA + treadmill (right) groups on day 14 post-stroke. (c) T2 hyperintense signal evolution from days 1 to 28. Stroke + rt-PA + treadmill *n* = 10 (female = 5, male = 5), stroke + rt-PA + no treadmill *n* = 10 (female = 5, male = 5). Changes in T2 hyperintense lesion volume were assessed using two-way repeated measures ANOVA with Sidak’s post hoc test. (d) Averaged time to contact (left) and time to removal (middle) from the adhesive removal test pre-stroke and on days 1, 3, 14 and 28. Stroke + rt-PA + treadmill *n* = 10 (female = 5, male = 5), stroke + rt-PA + no treadmill *n* = 10 (female = 5, male = 5). Right: Comparison of the discrimination index calculated from the NOR test pre-stroke to days 3, 14 and 28 post-stroke. Stroke + rt-PA + treadmill *n* = 10 (female = 5, male = 5), stroke + rt-PA + no treadmill *n* = 10 (female = 5, male = 5). Group differences across time were analyzed using two-way repeated measures ANOVA with Sidak’s post hoc test. (e) Representative immunofluorescent images showing selective neuronal death and reactive astrogliosis in the peri-infarct and contralateral areas on day 28 post-stroke. The first row shows ROIs for the stroke + rt-PA + no treadmill group. The second row shows ROIs for the stroke + rt-PA + treadmill group. NeuN-positive cells in the periinfarct area are shown in the first column. GFAP-positive cells in the periinfarct area are shown in the second column. Both NeuN and GFAP-positive cells in the contralateral area are shown in the third column. ROI (white box) example locations can be found in the graphics at the top of each column. (Top right) Number of NeuN positive cells in the peri-infarct area on day 28 post-stroke. Stroke + rt-PA + treadmill *n* = 5 (female = 3, male = 2), slices *n* = 20, ROIs *n* = 20; stroke + rt-PA + no treadmill *n* = 5 (female = 3, male = 2), slices *n* = 20, ROIs *n* = 20. (Bottom right) Reactive astrogliosis shown as GFAP fluorescence intensity in the peri-infarct area on day 28 post-stroke. Stroke + rt-PA + treadmill *n* = 5 (female = 3, male = 2), slices *n* = 20, ROIs *n* = 20; stroke + rt-PA + no treadmill *n* = 5 (female = 3, male = 2), slices *n* = 15, ROIs *n* = 15. Data were compared using two-tailed Mann–Whitney *U* test. *p < 0.05. ***p < 0.001. ****p < 0.0001.

Finally, we employed structural MRI and behavioural tests to examine the effect of cardiovascular exercise on T2 hyperintense signal evolution, sensorimotor and cognitive function (Figure 5(a) and (b)). The corrected T2 hyperintense volumes were similar in both treatment groups on day 1 (67.3 ± 42.7 mm^3^ vs 70.3 ± 34.8 mm^3^) and 4 (37.5 ± 26.4 mm^3^ vs 47.1 ± 26.8 mm^3^; Figure 5(c)). From day 4, T2 volumes steadily decreased in the no treadmill exercise group to day 28 (day 14: 35.3 ± 23.0 mm^3^; day 28: 26.7 ± 21.1 mm^3^; Figure 5(c)). Interestingly, treadmill exercise resulted in a steep decrease in T2 hyperintense lesion volume between days 4 and 14 (37.5 ± 26.4 mm^3^ to 14.8 ± 14.7 mm^3^), further decreasing to 10.4 ± 8.5 mm^3^ on day 28 (Figure 5(c)). T2 hyperintense lesion volumes in the treadmill exercise group were significantly smaller on days 14 (*p* = 0.0310) and 28 (*p* = 0.0436) than in the no treadmill exercise group, indicating an impact of cardiovascular exercise on edema and lesion evolution.

Smaller regions of T2 hyperintense signals in the treadmill exercise group were associated with less severe sensorimotor deficits on both days 14 (time to contact: 18.2 ± 4.9 s vs 27.6 ± 9.6 s, *p* = 0.0004 time to removal: 21.3 ± 6.3 s vs 34 ± 12.1 s, *p* = 0.0002) and 28 (time to contact: 12.5 ± 2.8 s vs 19.5 ± 6.4 s, *p* < 0.0001, time to removal 16.3 ± 5.4 s vs 25.1 ± 8 s, *p* = 0.0003; Figure 5(d)). Furthermore, after treadmill exercise rats showed significantly higher discrimination index scores in the NOR behavioural test on day 28 compared to those without treadmill exercise (0.133 ± 0.428 vs −0.301 ± 0.436, *p* = 0.0376; Figure 5(d)).

Next, we investigated selective neuronal death and reactive astrogliosis in the peri-infarct and contralateral areas on day 28 (Figure 5(e)). Animals that had undergone treadmill exercise had significantly higher numbers of NeuN-positive cells in the peri-infarct area, indicating more neuronal survival (Figure 5(e)). Moreover, GFAP signal intensity in the peri-infarct area was significantly higher in the no-treadmill exercise group indicating increased reactive astrogliosis and inflammation (Figure 5(e)).

Individual level correlation analyses confirmed a significant correlation between individual ASL perfusion values on day 14 and stroke outcomes (Supplemental Figure 1(a)). Specifically, higher ASL perfusion values were associated with smaller T2 signal, enhanced sensorimotor performance, and improved cognitive function. This trend persisted though day 28, with elevated ASL perfusion continuing to correlate with more favourable outcomes across these measures (Supplemental Figure 1(b)).

## Discussion

Reperfusion failure following stroke is increasingly recognized as a critical yet incompletely understood phenomenon. Using the thrombin model of stroke in rats – which allows for the formation of a real clot in situ, interaction with the endothelium, and thrombolytic treatment with rt-PA – this study offers valuable insights into the extent, potential mechanisms, and mitigation of reperfusion failure after stroke and IVT. While this model has been extensively characterized in mice,^25^ its application in rats has been more limited.^33^ Even though it requires craniotomy and arterial puncture, the risk of subarachnoid haemorrhage or other bleeding complications is minimal, and mortality was low in our study accordingly (6 out of 64 rats died prematurely throughout the experiments). The thrombin model’s use in rats enabled us to perform complex behavioural assessments, offering a robust foundation for translational stroke research.

Our findings highlight the significant effect of IVT while also revealing a persistent impairment in microvascular reperfusion despite successful recanalization of the occluded vessel. As expected, rats treated with rt-PA exhibited significantly higher levels of reperfusion and smaller infarct volumes compared to sham-treated stroke animals, consistent with previous reports using the same model in rodents.^9,25,26,33,34^ Although the effect of rt-PA on reperfusion was significant on day 1 and less pronounced on day 7, this early enhancement of CBF within the affected area might be sufficient to reduce infarct size on day seven. Nevertheless, despite successful thrombolysis and recanalization of the MCA, reperfusion only reached approximately 65% of baseline levels by the end of the LSCI recording. This finding confirms that the extent of reperfusion failure in rats mirrors that observed in mice in the acute phase of stroke.^9,26^

Several mechanisms have been implicated in this impaired microvascular reperfusion, including neutrophil stalls, gene expression changes compromising BBB integrity, loss of vascular tone, abnormal vessel constriction and capillary pericyte damage,^9,10,35–37^ Whilst most existing studies focus primarily on the acute and subacute phase post-stroke, our findings provide new insights into the persistence of these microvascular abnormalities. Specifically, we reveal that, although larger vessels regain functionality post-IVT, a significant chronic hypoperfusion persists, until at least day 28 after stroke, a previously underexplored facet of post-stroke pathology.

Further support for ongoing microvascular dysfunction comes from our immunofluorescence analyses, which implicates microclot formation and BBB damage as key contributors to inadequate tissue reperfusion. Despite improvements in outcomes post-stroke with rt-PA treatment, significant deficits and lesions persisted, highlighting the need for adjunctive therapeutic strategies to address reperfusion failure and the subsequent chronic hypoperfusion that follows.

It is well established that CVE benefits overall health, and in healthy individuals, it significantly increases CBF, with transcranial Doppler studies reporting an exponential rise in MCA velocity – ranging from 13.4 to 15.5 cm/s depending on the hemisphere – during moderate-intensity exercise, thereby enhancing oxygen and nutrient delivery to the brain.^38,39^ Multiple clinical studies have shown that CVE preconditioning not only lowers stroke risk but also reduces stroke severity and improves functional recovery.^40–42^ Moreover, post-stroke CVE has been associated with reduced lesion volumes, increased cell survival, and enhanced motor and cognitive functions when initiated early and at moderate intensity in rodents.^43–45^ Despite these insights, the impact of CVE on reperfusion failure following stroke remains largely unexplored. While preclinical studies in rodent models have demonstrated that CVE improves cerebral perfusion and promotes angiogenesis, its impact on the progression of reperfusion, particularly from the acute to chronic stages of stroke, has not been thoroughly investigated.^14,15,32,43^ Specifically, how CVE influences the persistence or resolution of microvascular reperfusion failure over time remains unclear.

In our study, we provide evidence that moderate CVE, initiated shortly after stroke, mitigates reperfusion failure and improves chronic hypoperfusion, and that this effect translates into smaller infarct lesions and better functional outcomes. Remarkably, perfusion in the lesion area exceeded baseline levels 12 days after exercise cessation, indicating a sustained and potentially compensatory vascular response. These benefits were more pronounced in stroke rats undergoing CVE compared to sham stroke rats receiving CVE, emphasizing the role of exercise in enhancing cerebral perfusion, mitigating reperfusion failure and supporting recovery after stroke.

These findings suggest that CVE may exert its effects on reperfusion in a phase-specific manner. During the sub-acute phase, exercise may alleviate reperfusion failure by enhancing vasodilation, improving endothelial function and BBB integrity, and increasing fibrinolytic activity.^46,47^ Mechanistically, this is supported by our observations that CVE reduced microclot burden and BBB leakage. Beyond the sub-acute stage, CVE is likely to support recovery through promotion of angiogenesis by inducing pro-angiogenic signals such as MT1-MMP and Tie-2.^14,32^ Accordingly, in our study, CVE led to increased vessel density in the peri-infarct area, which may improve reperfusion and long-term tissue repair. While our results strongly support a role of CVE in promoting long-term vascular remodelling and recovery, the timing, spatial distribution, and specific mechanisms of exercise-induced angiogenesis remain to be fully elucidated.

In individual animals, higher perfusion values assessed using ASL post-stroke correlated strongly with smaller T2 lesion volumes, improved sensorimotor performance and enhanced cognitive function, reinforcing the hypothesis that increased cerebral perfusion may support neuroplasticity and tissue repair. Importantly, these benefits persisted throughout the sub-acute stage, with increased vessel density and improved reperfusion evident up to 28 days post-stroke, suggesting that CVE may facilitate enduring changes in vascular architecture and functional connectivity.

Our findings align with the growing body of evidence that non-pharmacological interventions like CVE can serve as essential adjuncts to pharmacologic treatments for stroke.^14,15,32^ CVE is attractive from a translational perspective because it is widely accessible, generally safe, and can be integrated into rehabilitation programs. However, clinical data also highlight important caveats. The AVERT trial and follow-up studies, for example, reported that very early and intensive mobilization after stroke was associated with poorer outcomes, in contrast to the beneficial effects of post-stroke CVE observed in our rat model.^48^ These divergent results may reflect differences in timing and exercise dose, but also the inherent challenges of delivering and standardizing exercise-based interventions across diverse patient populations, clinical environments and countries.^49^ Future studies should therefore aim to define optimal parameters, such as intensity, timing and duration, for exercise interventions, potentially using an individualized approach to maximize recovery benefits.^50^

Finally, combining CVE with pharmacological agents that protect the BBB or modulate the inflammatory response could offer synergistic benefits, further reducing reperfusion failure and enhancing stroke recovery. Our findings provide a strong rationale for continued investigation into integrated therapeutic strategies and highlight the thrombin stroke model in rats as a valuable platform for such translational research.

## Supplemental Material

sj-docx-1-jcb-10.1177_0271678X251405677 – Supplemental material for Cardiovascular exercise mitigates reperfusion failure and persistent hypoperfusion in the thrombin model of stroke and thrombolysis Supplemental material, sj-docx-1-jcb-10.1177_0271678X251405677 for Cardiovascular exercise mitigates reperfusion failure and persistent hypoperfusion in the thrombin model of stroke and thrombolysis by William Middleham, Nadine F Binder, Julian Deseö, Robert Weber, Kirill Zolotko, Jeanne Droux, Hikari Yoshihara, Matthias T Wyss, Andreas R Luft, Bruno Weber, Daniel Razansky, Mohamad El Amki and Susanne Wegener in Journal of Cerebral Blood Flow & Metabolism

## References

[1] Mosconi MG ads Paciaroni M. Treatments in ischemic stroke: current and future. Eur Neurol 2022; 85(5): 349–366. 35917794 10.1159/000525822

[2] Hacke W Kaste M Bluhmki E , et al. Thrombolysis with alteplase 3 to 4.5 hours after acute ischemic stroke. N Engl J Med 2008; 359(13): 1317–1329. 18815396 10.1056/NEJMoa0804656

[3] Smith WS Sung G Saver J , et al. Mechanical Thrombectomy for acute ischemic stroke: final results of the multi MERCI trial. Stroke 2008; 39(4): 1205–1212. 18309168 10.1161/STROKEAHA.107.497115

[4] Guedin P Larcher A Decroix JP , et al. Prior IV thrombolysis facilitates mechanical thrombectomy in acute ischemic stroke. J Stroke Cerebrovasc Dis 2015; 24(5): 952–957. 25804567 10.1016/j.jstrokecerebrovasdis.2014.12.015

[5] Kloner RA King KS Harrington MG. No-reflow phenomenon in the heart and brain. Am J Physiol Heart Circ Physiol 2018; 315(3): H550–H562. 10.1152/ajpheart.00183.2018 29882685

[6] Amki ME Wegener S. Reperfusion failure despite recanalization in stroke: new translational evidence. Clin Trans Neurosci 2021; 5(1): 2514183X2110071.

[7] El Amki M Wegener S . Improving cerebral blood flow after arterial recanalization: a novel therapeutic strategy in stroke. IJMS 2017; 18(12): 2669. 29232823 10.3390/ijms18122669 PMC5751271

[8] Wong GJ Yoo B Liebeskind D , et al. Frequency, determinants, and outcomes of emboli to distal and new territories related to mechanical thrombectomy for acute ischemic stroke. Stroke 2021; 52(7): 2241–2249. 34011171 10.1161/STROKEAHA.120.033377

[9] El Amki M Glück C Binder N , et al. Neutrophils obstructing brain capillaries are a major cause of no-reflow in ischemic stroke. Cell Rep 2020; 33(2): 108260. 33053341 10.1016/j.celrep.2020.108260

[10] Erdener ŞE Tang J Kılıç K , et al. Dynamic capillary stalls in reperfused ischemic penumbra contribute to injury: a hyperacute role for neutrophils in persistent traffic jams. J Cereb Blood Flow Metab 2021; 41(2): 236–252. 32237951 10.1177/0271678X20914179 PMC8370003

[11] Jia M Jin F Li S , et al. No-reflow after stroke reperfusion therapy: an emerging phenomenon to be explored. CNS Neurosci Ther 2024; 30(2): e14631. 10.1111/cns.14631 PMC10867879 38358074

[12] Sperring CP Savage WM Argenziano MG , et al. No-reflow post-recanalization in acute ischemic stroke: mechanisms, measurements, and molecular markers. Stroke 2023; 54(9): 2472–2480. 37534511 10.1161/STROKEAHA.123.044240

[13] Yemisci M Gursoy-Ozdemir Y Vural A , et al. Pericyte contraction induced by oxidative-nitrative stress impairs capillary reflow despite successful opening of an occluded cerebral artery. Nat Med 2009; 15(9): 1031–1037. 19718040 10.1038/nm.2022

[14] Zhang P Yu H Zhou N , et al. Early exercise improves cerebral blood flow through increased angiogenesis in experimental stroke rat model. J NeuroEng Rehab 2013; 10(1): 43. 10.1186/1743-0003-10-43 PMC3648391 23622352

[15] Gao Y Zhao Y Pan J , et al. Treadmill exercise promotes angiogenesis in the ischemic penumbra of rat brains through caveolin-1/VEGF signaling pathways. Brain Res 2014; 1585: 83–90. 25148708 10.1016/j.brainres.2014.08.032

[16] Zhu A Lin Y Hu X , et al. Treadmill exercise decreases cerebral edema in rats with local cerebral infarction by modulating AQP4 polar expression through the caveolin-1/TRPV4 signaling pathway. Brain Res Bull 2022; 188: 155–168. 35961528 10.1016/j.brainresbull.2022.08.003

[17] Lu J Wang J Yu L , et al. Treadmill exercise attenuates cerebral ischemia–reperfusion injury by promoting activation of M2 microglia via upregulation of interleukin-4. Front Cardiovasc Med 2021; 8: 735485. 34692788 10.3389/fcvm.2021.735485 PMC8532515

[18] Heuschmann PU Wiedmann S Wellwood I , et al. Three-month stroke outcome: The European Registers of Stroke (EROS) investigators. Neurology 2011; 76(2): 159–165. 21148118 10.1212/WNL.0b013e318206ca1e

[19] Luft AR Macko RF Forrester LW , et al. Treadmill exercise activates subcortical neural networks and improves walking after stroke: a randomized controlled trial. Stroke 2008; 39(12): 3341–3350. 18757284 10.1161/STROKEAHA.108.527531 PMC2929142

[20] Hasan SMM Rancourt SN Austin MW , et al. Defining optimal aerobic exercise parameters to affect complex motor and cognitive outcomes after stroke: a systematic review and synthesis. Neural Plast 2016; 2016: 2961573. 26881101 10.1155/2016/2961573 PMC4736968

[21] Pang MYC Charlesworth SA Lau RWK , et al. Using aerobic exercise to improve health outcomes and quality of life in stroke: evidence-based exercise prescription recommendations. Cerebrovasc Dis 2013; 35(1): 7–22. 23428993 10.1159/000346075

[22] Patel H Alkhawam H Madanieh R , et al. Aerobic vs anaerobic exercise training effects on the cardiovascular system. World J Cardiol 2017; 9(2): 134–138. 28289526 10.4330/wjc.v9.i2.134 PMC5329739

[23] Querido JS Sheel AW. Regulation of cerebral blood flow during exercise. Sports Med 2007; 37(9): 765–782. 17722948 10.2165/00007256-200737090-00002

[24] Travers G Kippelen P Trangmar SJ , et al. Physiological function during exercise and environmental stress in humans-an integrative view of body systems and homeostasis. Cells 2022; 11(3): 383. 35159193 10.3390/cells11030383 PMC8833916

[25] Orset C Macrez R Young AR , et al. Mouse model of in situ thromboembolic stroke and reperfusion. Stroke 2007; 38(10): 2771–2778. 17702959 10.1161/STROKEAHA.107.487520

[26] Binder NF El Amki M Glück C , et al. Leptomeningeal collaterals regulate reperfusion in ischemic stroke and rescue the brain from futile recanalization. Neuron 2024; 112(9): 1456–1472.e6. 10.1016/j.neuron.2024.01.031 38412858

[27] Chen Z Zhou Q Droux J , et al. Transcranial cortex-wide imaging of murine ischemic perfusion with large-field multifocal illumination microscopy. Stroke 2025; 56(1): 170–182. 39705394 10.1161/STROKEAHA.124.047996

[28] Glück C Zhou Q Droux J , et al. Pia-FLOW: deciphering hemodynamic maps of the pial vascular connectome and its response to arterial occlusion. Proc Natl Acad Sci U S A 2024; 121(28): e2402624121. 10.1073/pnas.2402624121 PMC11252916 38954543

[29] Bouet V Boulouard M Toutain J , et al. The adhesive removal test: a sensitive method to assess sensorimotor deficits in mice. Nat Protoc 2009; 4(10): 1560–1564. 19798088 10.1038/nprot.2009.125

[30] Baumgartner P El Amki M Bracko O , et al. Sensorimotor stroke alters hippocampo-thalamic network activity. Sci Rep 2018; 8(1): 15770. 30361495 10.1038/s41598-018-34002-9 PMC6202365

[31] Antunes M Biala G. The novel object recognition memory: neurobiology, test procedure, and its modifications. Cogn Process 2012; 13(2): 93–110. 22160349 10.1007/s10339-011-0430-z PMC3332351

[32] Tang Y Zhang Y Zheng M , et al. Effects of treadmill exercise on cerebral angiogenesis and MT1-MMP expression after cerebral ischemia in rats. Brain Behav 2018; 8(8): e01079. 10.1002/brb3.1079 PMC6085910 30035384

[33] Arkelius K Vivien D Orset C , et al. Validation of a stroke model in rat compatible with rt-PA-induced thrombolysis: new hope for successful translation to the clinic. Sci Rep 2020; 10(1): 12191. 32699371 10.1038/s41598-020-69081-0 PMC7376012

[34] Ansar S Chatzikonstantinou E Wistuba-Schier A , et al. Characterization of a new model of thromboembolic stroke in C57 black/6J mice. Transl Stroke Res 2014; 5(4): 526–533. 24347404 10.1007/s12975-013-0315-9 PMC4092233

[35] Shrouder JJ Calandra GM Filser S , et al. Continued dysfunction of capillary pericytes promotes no-reflow after experimental stroke in vivo. Brain 2024; 147(3): 1057–1074. 38153327 10.1093/brain/awad401

[36] Hansen LMB Dam VS Guldbrandsen HØ , et al. Spatial transcriptomics and proteomics profiling after ischemic stroke reperfusion: insights into vascular alterations. Stroke 2025; 56(4): 1036–1047. 40052263 10.1161/STROKEAHA.124.048085

[37] Qiu B Zhao Z Wang N , et al. A systematic observation of vasodynamics from different segments along the cerebral vasculature in the penumbra zone of awake mice following cerebral ischemia and recanalization. J Cereb Blood Flow Metab 2023; 43(5): 665–679. 36524693 10.1177/0271678X221146128 PMC10108196

[38] Mulser L Moreau D. Effect of acute cardiovascular exercise on cerebral blood flow: a systematic review. Brain Res 2023; 1809: 148355. 37003561 10.1016/j.brainres.2023.148355

[39] Billinger SA Craig JC Kwapiszeski SJ , et al. Dynamics of middle cerebral artery blood flow velocity during moderate-intensity exercise. J Appl Physiol (1985) 2017; 122(5): 1125–1133. 28280106 10.1152/japplphysiol.00995.2016 PMC5451537

[40] Reinholdsson M Palstam A Sunnerhagen KS. Prestroke physical activity could influence acute stroke severity (part of PAPSIGOT). Neurology 2018; 91(16): e1461–e1467. 10.1212/WNL.0000000000006354 PMC6202943 30232251

[41] Willey JZ Moon YP Paik MC , et al. Physical activity and risk of ischemic stroke in the Northern Manhattan Study. Neurology 2009; 73(21): 1774–1779. 19933979 10.1212/WNL.0b013e3181c34b58 PMC2788811

[42] Armstrong MEG Green J Reeves GK , et al. Frequent physical activity may not reduce vascular disease risk as much as moderate activity: large prospective study of women in the United Kingdom. Circulation 2015; 131(8): 721–729. 25688148 10.1161/CIRCULATIONAHA.114.010296

[43] Austin MW Ploughman M Glynn L , et al. Aerobic exercise effects on neuroprotection and brain repair following stroke: a systematic review and perspective. Neurosci Res 2014; 87: 8–15. 24997243 10.1016/j.neures.2014.06.007

[44] Li F Geng X Huber C , et al. In search of a dose: the functional and molecular effects of exercise on post-stroke rehabilitation in rats. Front Cell Neurosci 2020; 14: 186. 32670026 10.3389/fncel.2020.00186 PMC7330054

[45] Hong M Kim M Kim TW , et al. Treadmill exercise improves motor function and short-term memory by enhancing synaptic plasticity and neurogenesis in photothrombotic stroke mice. Int Neurourol J 2020; 24(Suppl 1): S28–S38. 10.5213/inj.2040158.079 PMC7285698 32482055

[46] Małkiewicz MA Szarmach A Sabisz A , et al. Blood-brain barrier permeability and physical exercise. J Neuroinflamm 2019; 16(1): 15. 10.1186/s12974-019-1403-x PMC6345022 30678702

[47] Szymanski LM Pate RR. Fibrinolytic responses to moderate intensity exercise. Comparison of physically active and inactive men. Arterioscler Thromb 1994; 14(11): 1746–1750. 7947599 10.1161/01.atv.14.11.1746

[48] Langhorne P Wu O Rodgers H , et al. A very early rehabilitation trial after stroke (AVERT): a phase III, multicentre, randomised controlled trial. Health Technol Assess 2017; 21(54): 1–120. 10.3310/hta21540 PMC5641820 28967376

[49] Stoller O de Bruin ED Knols RH , et al. Effects of cardiovascular exercise early after stroke: systematic review and meta-analysis. BMC Neurol 2012; 12: 45. 22727172 10.1186/1471-2377-12-45 PMC3495034

[50] Rizvi MR Sharma A Malki A , et al. Enhancing cardiovascular health and functional recovery in stroke survivors: a randomized controlled trial of stroke-specific and cardiac rehabilitation protocols for optimized rehabilitation. J Clin Med 2023; 12(20): 6589. 37892727 10.3390/jcm12206589 PMC10607659

