# Supplementary material for: Cardiovascular exercise mitigates reperfusion failure and persistent hypoperfusion in the thrombin model of stroke and thrombolysis
Source: J Cereb Blood Flow Metab. 2026 Jan 16:0271678X251405677. Online ahead of print. doi: 10.1177/0271678X251405677 (PMC12811084; doi:10.1177/0271678X251405677)
Supplement: sj-docx-1-jcb-10.1177_0271678X251405677 – Supplemental material for Cardiovascular exercise mitigates reperfusion failure and persistent hypoperfusion in the thrombin model of stroke and thrombolysis [file sj-docx-1-jcb-10.1177_0271678X251405677.docx]

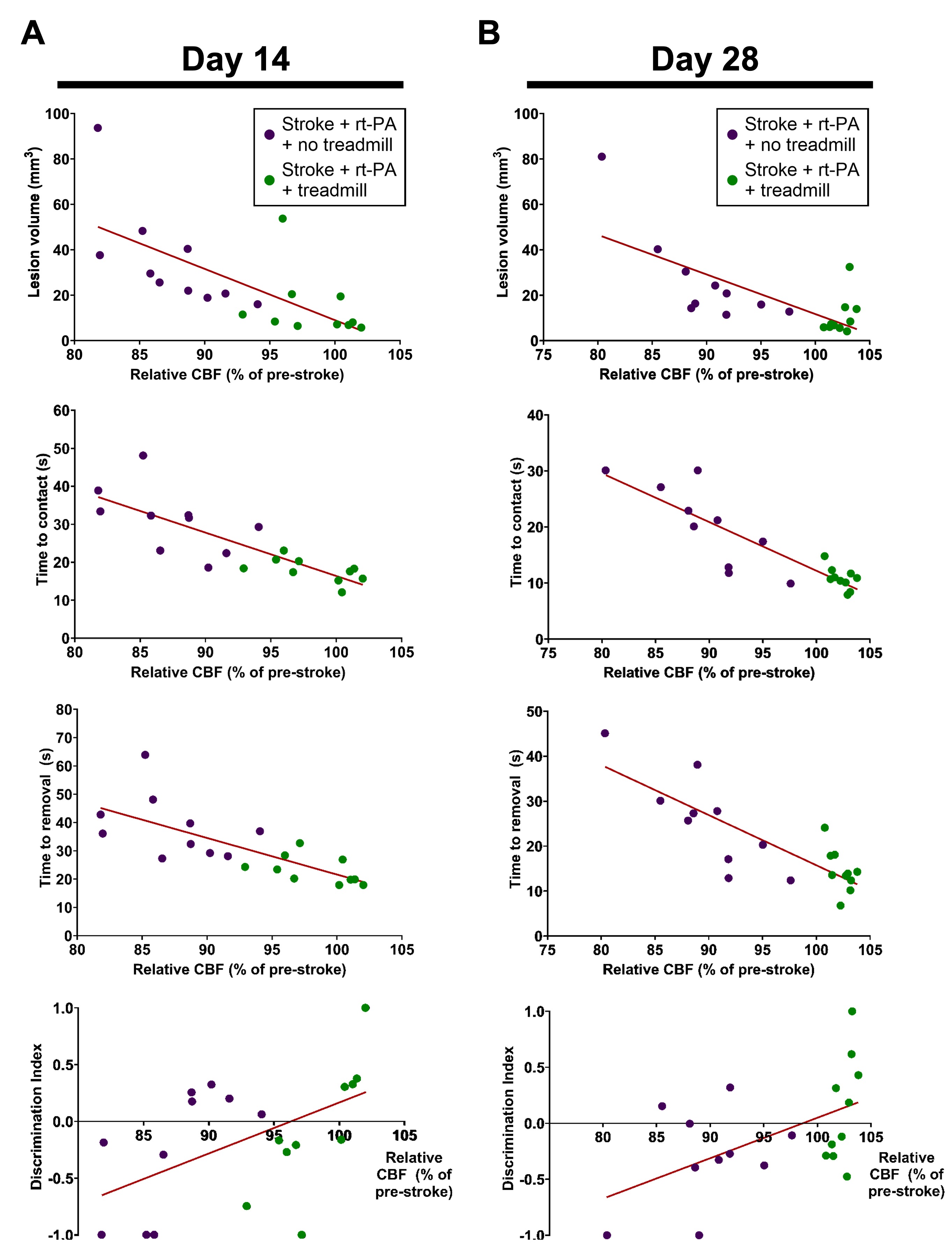


**Supplemental figure 1: Correlation analysis between brain perfusion measured by ASL imaging and stroke outcome parameters, related to Figures 4 and 5.**

(A) Correlation on day 14 post-stroke between ASL perfusion and T2 corrected lesion volume (Spearman r = -0.8000, p < 0.0001), time to contact (Spearman r = -0.877, p < 0.0001), time to removal (Spearman r = -0.8063, p < 0.0001) and discrimination index (Spearman r = 0.5759, p = 0.0079)

(B) Correlation on day 28 post-stroke between ASL perfusion and T2 corrected lesion volume (Spearman r = -0.5684, p = 0.0089), time to contact (Spearman r = -0.8304, p < 0.0001), time to removal (Spearman r = -0.7619, p < 0.0001), and discrimination index (Spearman r = 0.6138, p = 0.004)


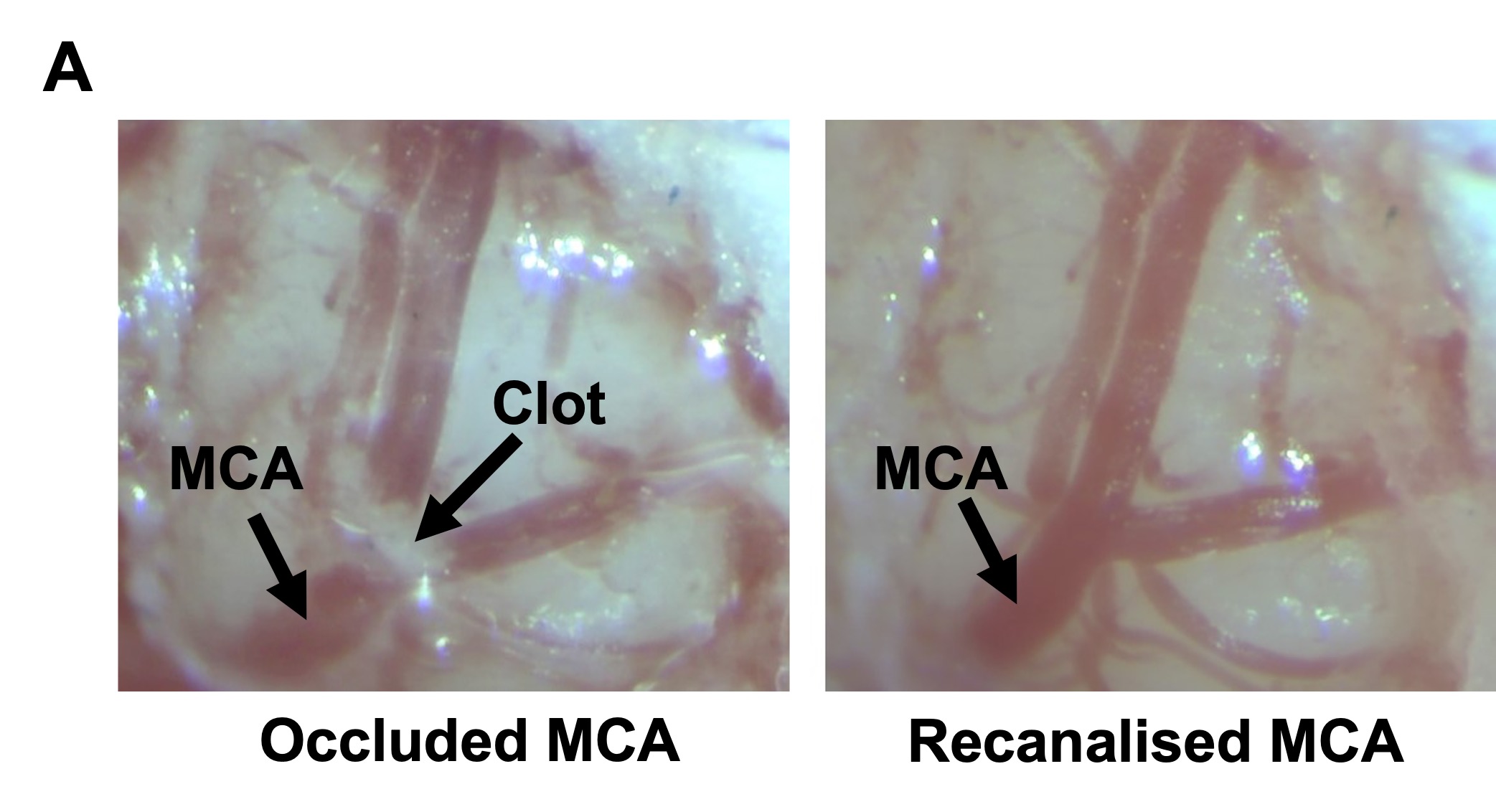


**Supplemental figure 2: Representative images of the occluded MCA by the clot (left) and the subsequent recanalized MCA after rt-PA treatment (right).**

**Supplemental table 1: Full statistical results for the two-way repeated measures ANOVA and Sidak’s post hoc test analyses.**

| **Figure** | **Measure** | **Main Effects & Interaction (AVOVA)** | **Post hoc (Sidak’s multiple comparisons)** |
| --- | --- | --- | --- |
| Figure 1F | LSCI | Time: F(2.225,42.27)=141.8, p<0.0001;  Group: F(1,19)=6.903, p = 0.0166;  Interaction: F(9,171)=8.67, p<0.0001 | 0 minutes: saline vs rt-PA ns.  10 minutes: saline vs rt-PA ns.  20 minutes: saline vs rt-PA ns.  30 minutes: saline vs rt-PA ns.  40 minutes: saline vs rt-PA ns.  50 minutes: saline vs rt-PA ns.  60 minutes: saline<rt-PA (p=0.0002).  70 minutes: saline<rt-PA (p=0.0029).  80 minutes: saline<rt-PA (p=0.0060).  90 minutes: saline<rt-PA (p<0.0001). |
| Figure 2C | ASL perfusion | Time: F(1.636,29.45)=107.8, p<0.0001;  Group: F(1,18)=7.178, p=0.0153  Interaction: F(2,36)=2.138, p=0.1326 | Baseline: no significant differences.  Day 1: saline<rt-PA (p=0.0257).  Day 7: saline vs rt-PA ns. |
| Figure 2D | Hypercapnic test | Time: ns (p=0.9071); Group: F(1,17)=24.70 (p=0.0001);  Interaction: F(2,34)=4.587, p=0.0172 | Baseline: no significant differences.  Day 1: saline<rt-PA (p=0.0004).  Day 7: saline<rt-PA (p=0.0044). |
| Figure 3C | Corrected T2 lesion volume | Time: F(1,18)=167.9, p<0.0001;  Group: F(1,18)=26.97, p<0.0001;  Interaction: ns (p=0.9668) | Day 1: saline>rt-PA (p<0.0001).  Day 7: saline>rt-PA (p<0.0001). |
| Figure 3E | Adhesive removal: Time to contact | Time: F(1.221,36.62)=88.96, p<0.0001;  Group: F(1,30)=35.77, p<0.0001;  Interaction: F(2,60)=19.03, p<0.0001 | Baseline: no significant differences.  Day 1: rt-PA<saline (p<0.0001).  Day 7: rt-PA<saline (p<0.0001). |
| Figure 3E | Adhesive removal: Time to removal | Time: F(1.364,40.93)=131.1, p<0.0001;  Group: F(1,30)=41.24 (p<0.0001);  Interaction: F(2,60)=17.85, p<0.0001 | Baseline: no significant differences.  Day 1: rt-PA<saline (p<0.0001).  Day 7: rt-PA<saline (p<0.0001). |
| Figure 3E | NOR discrimination index | Time: F(1,18)=54.35, p<0.0001;  Group: F(1,18)=5.961, p=0.0252;  Interaction: ns, p=0.6551 | Baseline: no significant differences.  Day 7: saline vs rt-PA ns. |
| Figure 4B | ASL perfusion (stroke + treadmill vs stroke + no treadmill) | Time: F(2.992,53.85)=71.12, p<0.0001;  Group: F(1,18)=31.86, p<0.0001;  Interaction: F(4,72)=17.66, p<0.0001 | Baseline: no significant differences.  Day 1: stroke + treadmill vs stroke + no treadmill ns.  Day 4: stroke + treadmill vs stroke + no treadmill ns.  Day 14: stroke + treadmill>stroke + no treadmill (p<0.0001).  Day 28: stroke + treadmill>stroke + no treadmill (p<0.0001). |
| Figure 4B | ASL perfusion (sham + treadmill vs sham + no treadmill) | Time: F(1.833,12.83)=5.388, p=0.0219;  Group: F(1,7)=6.400, p=0.0392;  Interaction: F(4,28)=6.157, p=0.0011 | Baseline: no significant differences  Day 1: sham + treadmill vs sham + no treadmill ns  Day 4: sham + treadmill vs sham + no treadmill ns  Day 14: sham + treadmill vs sham + no treadmill ns  Day 28: sham + treadmill>sham + no treadmill (p=0.0019). |
| Figure 4C | Hypercapnic test (stroke + treadmill vs stroke + no treadmill) | Time: ns (p=0.3436); Group: ns (p=0.7648); Interaction: ns (p=0.1665) | No significant differences at any timepoint. |
| Figure 4C | Hypercapnic test (sham + treadmill vs sham + no treadmill) | Time: ns (p=0.6047);  Group: ns (p=0.6771);  Interaction: ns (p=0.9896) | No significant differences at any timepoint. |
| Figure 5C | Corrected T2 lesion volume | Time: F(1.129,20.33)=66.95, p<0.0001;  Group: F(18,54)=15.92, p<0.0001;  Interaction: ns (p=0.1323) | Stroke + treadmill<stroke + no treadmill (Day 14: p=0.0310; Day 28: p=0.0436). No differences Days 1 or 4. Both groups decreased over time. |
| Figure 5D | Adhesive removal: Time to contact | Time: F(1.591,60.45)=85.35, p<0.0001;  Group: F(1,34)=5.559, p=0.0243;  Interaction: ns (p=0.3362) | Both groups impaired vs baseline (p<0.0001).  Day 1 and Day 3: stroke + treadmill vs stroke + no treadmill ns (p=0.8648 and p=0.5895).  Day 14 and Day 28: stroke + treadmill<stroke + no treadmill (p=0.0004 and p<0.0001). |
| Figure 5D | Adhesive removal: Time to removal | Time: F(1.723,65.49)=109.9, p<0.0001;  Group: F(1,31)=4.974, p=0.0331;  Interaction: ns (p=0.0519) | Both groups impaired vs baseline (p<0.0001).  Day 1 and Day 3: stroke + treadmill vs stroke + no treadmill ns (p=0.9049 and p=0.8526).  Day 14 and Day 28: stroke + treadmill<stroke + no treadmill (p=0.0002 and p=0.0003). |
| Figure 5D | NOR discrimination index | Time: F(2.593,46.67)=6.988, p=0.0010;  Group: ns (p=0.1663);  Interaction: ns (p=0.2306) | Baseline: no differences.  Day 3: stroke + treadmill vs stroke + no treadmill ns (p=0.4134).  Day 14: stroke + treadmill vs stroke + no treadmill ns (p=0.4601).  Day 28: stroke + treadmill exercise>stroke + no treadmill (p=0.0376).  Within no treadmill: significant decline from baseline at day 14 and 28, p=0.0205 and p=0.0166). |
